# Acute and sub-acute toxicity study of a Pakistani polyherbal formulation

**DOI:** 10.1186/s12906-017-1889-7

**Published:** 2017-08-04

**Authors:** Saiqa Ishtiaq, Maida Akram, Sairah Hafeez Kamran, Uzma Hanif, Muhammad Shaharyar Khan Afridi, Sajid-ur-Rehman, Atika Afzal, Ayesha Asif, Muhammad Younus, Shehla Akbar

**Affiliations:** 1 Punjab University College of Pharmacy, University of the Punjab Allama Iqbal Campus, Lahore, 54000 Pakistan; 2 Department of Botany, Government College University, Lahore, Pakistan; 3 Department of Pharmacy, Islamia University of Bahawalpur, Bahawalpur, Pakistan; 4 Department of Pharmacy, Hajvery University, Lahore, Pakistan; 5 Lahore College of Pharmaceutical Sciences, Lahore, Pakistan

**Keywords:** Toxicity studies, Polyherbal drug, Herbology, Acute toxicity, Sub-acute toxicity, Complementary medicine

## Abstract

**Background:**

Herbology is the prevailing system among the nationally-accepted alternative or complementary systems of medicine. The system is due to its general and patient-oriented methodology, is widely used in the general population exposing them to the risk of the side effects of the herbal medicines.

**Method:**

The aim of study was to assess the acute and sub-acute toxicity of the polyherbal formulation Hab-e-Kabad Noshadri tablets. In the acute arm of the study, a single dose of 2000 mg/kg was administered to Swiss Albino mice which were observed for physical symptoms and behavioral changes for 72 h. In sub-acute toxicity study repeated doses of the polyherbal preparation was administered to Wistar rats of both genders, separately. The animals received three doses of polyherbal product (50 mg/kg/day, 100 mg/kg/day and 200 mg/kg/day) for a period of 28 days. On 28th day of experiment, blood sampling of animals was done for hematological and biochemical analysis i.e. liver and renal function parameters, lipid profile and then sacrificed for histopathological examination of liver and kidney.

**Result:**

There was no morbidity and mortality noticed with single dose administration in acute toxicity study in mice. In sub-acute toxicity study, morphological changes with some damage in liver and kidney tissues of male and female animals were recorded at dose of 100 mg/kg/day and 200 mg/kg/day.

**Conclusions:**

It was found that prolonged use at higher dose i.e. 200 mg/kg/day of this polyherbal formulation should be avoided and practitioners should cautiously prescribe this formulation in patients with hepatic and renal impairment.

**Electronic supplementary material:**

The online version of this article (doi:10.1186/s12906-017-1889-7) contains supplementary material, which is available to authorized users.

## Background

Herbal medicines are the most popular form of therapy for most of the world’s population. A large number of population in the developing countries still rely on herbal medicine practitioners to meet their primary healthcare needs. The major reasons behind utilization of herbal medicines are that they are affordable, easily accessible, patient oriented and closely relates to patient’s belief. This practice being natural and safe, perceived as non-toxic by the general population [1]. In the eighteenth century, when the medicinal therapy era was being introduced, the herbal treatment was the most preferred and available therapy. Many compounds from herbal origin have achieved widespread use as medicinal agents e.g. Taxols from *Taxus baccata* (English yew) as anticancer agents*,* Silibinin from *Silybum marianum* (Milk thistle) as liver tonic *etc.* [2–4]*.* The herbal therapy encompasses Ayurvedic, Naturopathic, Biochemical, Unani, Chinese, African and Native American medicine [2, 5]. Herbal medicines have attained the widespread acceptability as natural therapeutic agents for various diseases like diabetes, arthritis, renal and liver diseases, obesity and cardiovascular disorders [6].

In contrast to the breakthrough of various conventional drugs from the studies of traditional cures and folk knowledge, a number of botanical drugs have proved to be very efficient in the management of various sicknesses. For example; *Digitalis* (Foxglove) as cardiotonic for heart failure, *Catharanthus* (Rozy periwinkle) as anticancer, *Pilocarpus* to treat dry mouth and glaucoma, *Glycyrrhiza* (liquorice) and *Commiphora* (Myrrh) for cough, *Allium sativum* (Garlic) as cholesterol lowering agent *etc.* [7, 8]. Around 70% of the local public relies totally on phytomedicines, they have clearly recurred into the setting as the need of the hour in primary healthcare [9]. Oriental herbology is mostly carried out, in which combination of different herbs are made to achieve maximum therapeutic outcomes than the individual herbs. These combinations are employed for the betterment of various chronic disorders [10–12] e.g. Diakyur, (polyherbal drug) employed in the management of type II diabetes, contains seven herbal materials [13]. Similarly, Soshiho-tang, consist of seven herbs, is an oriental herbal formula used in Japan, China and Korea. It possesses various pharmacological actions that includes; antioxidant, anti-inflammatory, immunomodulatory, anti-hepatic fibrotic, hepatoprotective, and antitumoral effects [14].

The pharmacological aspects and properties of these drugs have been studied, by various researchers in different laboratories, extensively. In addition to this, conventional people and even still certain physicians invoke the usage of medicinal and curative herbs which are well documented with scientific evidences, to aid the medication therapy for better clinical outcomes but their selection is limited not only due to concerns of pharmaceutical quality but also in terms of their safety profile and toxicity index [15]. In spite of huge usefulness of these herbal remedies, there are certain limitations to their use. There is a lack of data on their safety and toxicity index. Certain toxicity studies has captured the attention and compelled the researchers to work and evaluate the safety spectrum of these phytomedicines [16, 17].

The test preparation is one of a renowned polyherbal natural formulation in Pakistan. The product label claims it to be an effective medicine for the treatment of hepatitis, enlargement of liver (hepatomegaly), liver function disorders, dyspepsia (indigestion), flatulence (abdominal gas), abdominal pain, nausea, vomiting, lack of appetite, constipation and carminative. The dose of the formulation as claimed by the label is 3 g/day for adults and 1.5 g/day for children [18]. As the exact duration of use and the side effects of the product is not mentioned on the label, we designed this study to assess the safety profile and toxicity index of the polyherbal formulation for a specific period of time.

## Methods

### Herbal product

The Hab-e-Kabad Noshadri tablets is a product of Qarshi Industries (Pvt.) Ltd. The product contains Ammonium chloride (17.55 mg), Black salt (17.55 mg), Common salt (NaCl) (17.55 mg), Lake salt (17.55 mg), Zedoary *(Curcuma zedoaria)* (17.55 mg)*,* Sodium borate (17.55 mg), Ginger *(Zingiber officinale)* (17.55 mg), Chebulicmyrobalan black *(Terminalia chebula)* (17.55 mg), Embelia *(Embelia ribs)* (17.55 mg), Rose *(Rosa indica)* (17.55 mg), Belericmyrobalan *(Terminalia belerica)* (17.55 mg), Black pepper *(Piper nigrum)* (17.55 mg), Senna leaflets *(Cassia angustifolia, Cassia acutifolia)* (17.55 mg), and Chebulicmyrobalan yellow *(Terminalia chebula)* (35.10 mg).

### Experimental animals

Young healthy Swiss albino mice (both sexes), 5–6 weeks old, weighing about 24–25 g and Wistar rats (both sexes), 9–12 weeks old weighing about 150–200 g were used in this study. The animals were purchased from University of Veterinary and Animal Sciences Lahore, Pakistan. The animals were maintained under standard environmental conditions (23–25 °C, 12 h/12 h light/dark cycle) and had free access to standard pelleted diet, water *ad libitum*. Animals were acclimatized to laboratory environment for a week prior to start study. The protocol used in this study was approved by the Animal Ethical Committee of Punjab University College of Pharmacy (AEC/UCP/1043), prepared by National Institute of Health.

### Experimental design

#### Dose calculation

The different doses selected for the toxicity study were chosen on the basis of the claim of the polyherbal formulation label (3 g/day for adults). The 50 mg/kg/day was selected according to the dose employed in adult human beings on daily bases. Higher doses (100 and 200 mg/kg/day) were selected for sub-acute toxicity study. The dose of the individual rats in all different groups was calculated based on the body weights before the start of the study.

#### Acute toxicity study test

Mice were fasted for 24 h prior to the commencement of this test. Ten animals (mice); five males and five females were used and each animal were given a single dose of 2000 mg/kg of Polyherbal product (p.o.). Animals were observed strictly and individually for first 30 min after dosing and periodically during first 24 h. (with special attention during first 4 h) and daily thereafter for 3 days. Mice were observed for altered autonomic effects (lacrimation, salivation, piloerection), central nervous system effect (tremors, convulsion, drowsiness) skin (fur), body weight, food consumption, water consumption and mortality [19].

#### Sub-acute toxicity study

Forty Wistar rats were divided into 4 groups of 10 each (5 males & 5 females). Three groups of experimental doses 50 mg/kg/day, 100 mg/kg/day and 200 mg/kg/day respectively and the forth group was of control. Control group was fed with only normal food and water. Animals were weighed weekly and observed for behavioral changes, food and water consumption, and general morphological changes. On the 28th day of treatment period, animals were anaesthetized by i.p. administration of 5 ml/kg of a solution of 1% chloralose in 25% urethane (*w*/*v*). Blood samples were collected from rats by cardiac puncture into EDTA sample tubes for hematological analysis and into heparinized tubes for serum generation for biochemical analysis. Serum was acquired after allowing blood to congeal for 30 min. And centrifugation. After sacrificing the experimental animals, vital organs including; kidneys and Liver were harvested, carefully examined [20]. The offcuts of the organs were conserved for histopathological assessment. Mortality in each treatment group was recorded during the course of the 28 day administration of the product.

### Hematological analysis

Blood samples were analyzed by using established procedures and the CBC machine by **Medonic**. Parameters evaluated include; WBCs count, RBCs count, PLT count, Hb, HCT, MCV, MCH and MCHC.

### Biochemical parameters

Serum samples were analyzed for creatinine, blood urea, uric acid, TG, cholesterol, HDL, VLDL, bilirubin, AST and ALT by using instrument **Junior Series**.

### Histopathological assessment

Liver and kidney tissues were obtained from experimental animals and fixed in 10% formol-saline. Later, these tissues were dehydrated in graded alcohol, inserted in paraffin, and cut into 4–5 μm thick sections. Hematoxylin-eosin was used to stain the sections for photomicroscopic assessment using a Model N-400ME photomicroscope. Slides were examined using the 40X, and 100X objectives [21, 22].

### Statistical analysis

Results are communicated as mean ± SEM. Data analysis was carried out using One-way ANOVA with post-hoc Tukey’s HSD test. For weight variation, Two-way ANOVA with post-hoc Dunnett's test was applied for multiple comparisons (SPSS 21). Significance was measured at values of *p <* 0.05 and *p <* 0.01.

## Results

### Acute toxicity study

In acute toxicity testing, all animals were observed carefully for development of any toxic signs or symptoms at different time intervals of 0, 30 min, 1, 2, 4, 6, 8, 12 h. and then daily for a period of 3 days. There was no toxic signs observed in clinical parameters during acute study. So, it indicates that the LD_50_ of the polyherbal formulation is greater than 2000 mg/kg/day BW (Table 1).

**Table 1:** Acute study with dose of 2000 mg/kg/day on male and female mice for three days

Sr. No.	Parameters	Animals									
		Male 1	Male 2	Male 3	Male 4	Male 5	Female 1	Female 2	Female 3	Female 4	Female 5
1	Lacrimation	No	No	No	No	No	No	No	No	No	No
2	Salivation	No	No	No	No	No	No	No	No	No	No
3	Piloerection	No	No	No	No	No	No	No	No	No	No
4	Drowsiness	No	No	No	No	No	No	No	No	No	No
5	Tremors	No	No	No	No	No	No	No	No	No	No
6	Convulsions	No	No	No	No	No	No	No	No	No	No
7	Fur	Normal	Normal	Normal	Normal	Normal	Normal	Normal	Normal	Normal	Normal
8	Body weight	23 g	23 g	25 g	25 g	27 g	23 g	22 g	23 g	24 g	24 g
9	Food consumption	Normal	Normal	Normal	Normal	Normal	Normal	Normal	Normal	Normal	Normal
10	water consumption	Normal	Normal	Normal	Normal	Normal	Normal	Normal	Normal	Normal	Normal
11	Mortality	No	No	No	No	No	No	No	No	No	No

### Sub-acute toxicity study

The study was conducted for four weeks (28 days) with three different doses; 50 mg/kg/day, 100 mg/kg/day, 200 mg/kg/day and one group treated as control. The parameters focused were body weight, food and water consumption, hematological parameters (WBCs, RBCs, PLT, Hb, HCT, MCH, MCV, and MCHC), liver function parameters (Bilirubin, ALT, AST), renal function profile (blood urea, creatinine, and uric acid), and lipid profile (triglycerides, cholesterol, HDL, VLDL).

#### Effect on body weight

**Fig. 1:**
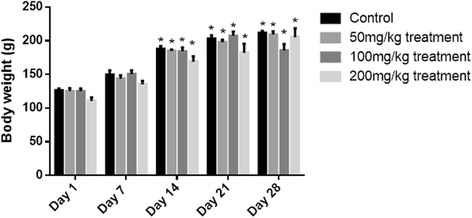
Body weight variation of control and treated male rats during sub-acute toxicity studies. The data is presented as mean ± SEM. Two-way ANOVA was used for multiple comparisons by applying Dunnett's test and the weights of all the groups were compared with their relevant weight on Day 1. * represents significance of *p <* 0.05

**Fig. 2:**
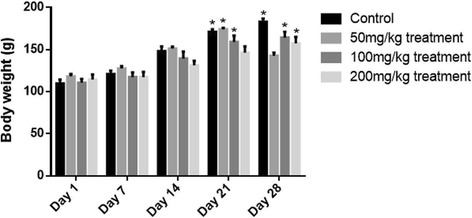
Body weight variation of control and treated female rats during sub-acute toxicity studies. The data is presented as mean ± SEM. Two-way ANOVA was used for multiple comparisons by applying Dunnett's test and the weights of all the groups were compared with their relevant weight on Day 1. * represents significance of *p <* 0.05

Weight variations of both male and female treated and control groups were noted. A gradual raise in the weights of male rats throughout the sub-acute study. There was a significant increase in the weights observed from day 14 till the end of the study in all treatment groups in comparison to their relevant weights on day 1 (Fig. 1). Whereas, for females a different pattern was observed. The graphical representation shows that at the start of the study the body weights of the treated groups were normal, no significant change in the weight was observed till day 14 as compared to their relevant weights on day 1. On day 21 there was a significant increase in weight of control, 50 and 100 mg/kg/day treatment groups but there was not any marked change for treatment group of 200 mg/kg/day. While on day 28, all groups i.e. control, 100 and 200 mg/kg/day except 50 mg/kg/day showed significant changes in weights for female rats (Fig. 2).

### Clinical signs

Nasal bleeding was observed on 17th and 18th day at dose of 200 mg/kg/day in both male and female rats. Paralysis in the right upper paw in both male and female group was observed 15 days after the treatment during sub-acute study at dose of 200 mg/kg/day (Table 2).

**Table 2:** Observations of physical signs during sub-acute toxicity study

Parameters	Males				Females			
	Control	50 mg/kg/day	100 mg/kg/day	200 mg/kg/day	Control	50 mg/kg/day	100 mg/kg/day	200 mg/kg/day
Nasal bleeding	−	−	−	+	−	−	−	+
Paralysis	−	−	−	+	−	−	−	+

*n* = 5, **−** = absence of the clinical signs, **+** = presence of the clinical signs

#### Effect on hematological parameters

The Hematological result shows that WBCs count of male animal of treatment dose of 100 mg/kg/day raise significantly and highly significantly for 200 mg/kg/day as that of control group. RBCs count was normal with all treatment groups as that of control. PLT count with treatment doses of 50 mg/kg/day and 100 mg/kg/day didn’t show any significant variation with that of control but with dose of 200 mg/kg/day PLT count showed highly significant increase with that of control. Hemoglobin value for dose of 200 mg/kg/day declined significantly as compared to control while remained near to control for rest of the treatment groups. HCT, MCV, MCH, MCHC remained normal. The Hematological estimation of female rats of all treatment doses and control group shows that there is no significant change in all parameters of complete blood count, but only the PLT count with the treatment dose of 200 mg/kg/day showed highly significant decrease with that of control but within the normal range [23] (Table 3; Additional file 1).

**Table 3:** Effect of different test doses of product on hematological parameters in male & female rats

Parameters	Males				Females			
	Control	50 mg/kg/day	100 mg/kg/day	200 mg/kg/day	Control	50 mg/kg/day	100 mg/kg/day	200 mg/kg/day
WBCs (10*3/ul)	4.91 ± 0.245	4.97 ± 0.141	5.79* ± 0.144	6.14** ± 0.143	2.642 ± 0.137	3.102 ± 1.090	3.350 ± 1.174	3.232 ± 1.128
RBCs (10*6/ul)	6.80 ± 0.178	6.33 ± 0.152	7.18 ± 0.141	6.28 ± 0.212	7.596 ± 0.83	7.214 ± 0.210	7.870 ± 0.197	7.544 ± 0.227
PLT (10*3/ul)	643.804 ± 2.429	644.09 ± 1.432	644.496 ± 1.373	654.26** ± 1.314	682.582 ± 1.928	675.50 ± 1.496	683.794 ± 1.941	667.068** ± 1.594
Hb (g/dl)	12.028 ± 0.218	12.038 ± 0.166	11.076 ± 0.143	10.972* ± 0.306	12.55 ± 0.240	12.66 ± 0.278	13.156 ± 0.306	13.052 ± 0.370
HCT (%)	40.066 ± 0.437	40.422 ± 0.338	40.172 ± 0.473	39.962 ± 0.337	38.276 ± 0.395	37.696 ± 0.505	38.726 ± 0.433	38.33 ± 0.561
MCV(fl)	76.020 ± 0.443	75.456 ± 0.432	75.728 ± 0.362	75.582 ± 0.310	74.302 ± 0.440	73.822 ± 0.532	73.926 ± 0.850	75.094 ± 0.820
MCH (pg)	20.788 ± 0.454	20.396 ± 0.289	20.242 ± 0.336	20.332 ± 0.371	21.716 ± 0.535	21.168 ± 0.295	21.048 ± 0.325	21.168 ± 0.567
MCHC (g/dl)	40.368 ± 0.400	40.610 ± 0.244	40.544 ± 0.237	40.468 ± 0.355	41.142 ± 0.358	41.574 ± 0.527	41.404 ± 0.621	41.126 ± 0.452

The data are expressed as mean and standard error of the mean (*n* = 5). For statistical analysis one-way ANOVA with post-hoc Tukey’s test was applied. * represents significance of *p <* 0.05, ** represents significance of *p <* 0.01

### Effect on biochemical parameters

#### Liver parameters

The liver parameters of male and female rats of all treatment doses groups showed that the levels of ALT and AST declined highly significantly as compared to control at dose of 200 mg/kg/day. Though with dose 50 and 100 mg/kg/day, the liver enzymes were near to the lower normal range as compared to the control group (Table 4).

**Table 4:** Effect of different test doses of product on bilirubin, ALT and AST in male and female rats

Parameters	Males				Females			
	Control	50 mg/kg/day	100 mg/kg/day	200 mg/kg/day	Control	50 mg/kg/day	100 mg/kg/day	200 mg/kg/day
Bilirubin (mg/dl)	1.575 ± 0.051	0.592** ± 0.011	0.602** ± 0.010	0.696** ± 0.013	1.0 ± 0.063	0.64** ± 0.045	0.76 ± 0.045	1.56** ± 0.060
ALT (μ/ml)	38.42 ± 0.229	8.4** ± 0.172	8.86** ± 0.141	6.942** ± 0.102	38.47 ± 0.309	9.54** ± 0.454	9.41** ± 0.383	9.28** ± 0.311
AST (μ/ml)	39.9 ± 0.251	10.0** ± 0.254	8.68** ± 0.154	8.62** ± 0.261	37.92 ± 0.419	10.98** ± 0.503	9.54** ± 0.310	8.98** ± 0.661

The data are expressed as mean and standard error of the mean (*n* = 5). For statistical analysis one-way ANOVA with post-hoc Tukey’s test was applied. * represents significance of *p <* 0.05, ** represents significance of *p <* 0.01

### Kidney parameters

Kidney parameters of male and female rats of both treatment and control groups showed that blood urea of the drug with dose of 50 mg/kg/day remained almost same as that of control. While the blood urea with the treatment doses of 100 mg/kg/day and 200 mg/kg/day were raised highly significantly with that of control but remained in the normal range. Creatinine level of male rats with dose of 50 mg/kg/day rose highly significantly with that of control but was in normal range while remained same with the treatment doses 100 mg/g/day and 200 mg/kg/day as that of control group. While, creatinine level of female rats with dose of 50 mg/kg/day rose highly significantly with that of control while with the treatment dose of 100 mg/kg/day didn’t show any change with that of control group and with dose of 200 mg/kg/day the creatinine level significantly decreased as that of control group. Result shows that uric acid level of male rats treatment doses of 50 mg/kg/day and 100 mg/kg/day has declined highly significantly but remained in the normal range as that of control but the treatment dose of 200 mg/kg/day remained almost same with that of control. On the other hand, in the female rats the uric acid level of treatment dose of 50 mg/kg/day didn’t show any noteworthy change with that of control group and with doses of 100 mg/kg/day and 200 mg/kg/day increased highly significantly as that of control (Table 5).

**Table 5:** Effect of different test doses of product on blood urea, creatinine and uric acid in male and female rats

Parameters	Males				Females			
	Control	50 mg/kg/day	100 mg/kg/day	200 mg/kg/day	Control	50 mg/kg/day	100 mg/kg/day	200 mg/kg/day
Blood urea (mg/dl)	23.6 ± 0.265	23.7 ± 0.432	28.7** ± 0.355	31.4** ± 0.966	22.98 ± 0.323	24.50 ± 0.295	28.75** ± 0.439	38.22** ± 0.461
Creatinine (mg/dl)	0.494 ± 0.029	0.686** ± 0.020	0.472 ± 0.036	0.428 ± 0.042	0.40 ± 0.029	0.652** ± 0.027	0.44 ± 0.022	0.29* ± 0.004
Uric acid (mg/dl)	3.476 ± 0.035	2.14** ± 0.223	2.4** ± 0.041	3.36 ± 0.137	3.44 ± 0.035	3.42 ± 0.033	3.98** ± 0.033	4.06** ± 0.045

The data are expressed as mean and standard error of the mean (*n* = 5). For statistical analysis one-way ANOVA with post-hoc Tukey’s test was applied. * represents significance of *p <* 0.05, ** represents significance of *p <* 0.01

### Lipid profile

The result of lipid profile of male rat treatment groups and control group shows that triglyceride level of treatment dose 200 mg/kg/day of the drug increased significantly as compared to control while in rest of the treatment doses the level remained close to the control. The cholesterol level of treatment doses of 50 mg/kg/day and 100 mg/kg/day showed highly significant increase with that of control group and treatment dose of 200 mg/kg/day showed highly significant decrease with that of control. HDL value with all treatment groups highly significantly decreased with that of control. VLDL value was normal with that of control with doses of 50 mg/kg/day and 100 mg/kg/day while with dose of 200 mg/kg/day VLDL value increased highly significantly. In case of females, animals showed that triglyceride level of treatment doses of 50 mg/kg/day and 200 mg/kg/day increased highly significantly with that of control but remained in safe range. With treatment dose of 100 mg/kg/day of the drug the triglyceride level remained in the range of control group. The cholesterol level of treatment dose of 50 mg/kg/day didn’t show any significant change with that of control. With dose of 100 mg/kg/day the cholesterol level raised highly significantly as compared to control group. With the higher treatment dose of 200 mg/kg/day the cholesterol level decreased highly significantly as compared to the control group. HDL value with all treatment groups highly significantly decreased with that of control. VLDL value was normal with that of control with doses of 50 mg/kg/day and 100 mg/kg/day. While, with dose of 200 mg/kg/day VLDL value increased highly significantly. All lipid profiles of male and female rats showed that the values were either significantly increased or decreased but remained within the normal range (Table 6).

**Table 6:** Effect of different test doses of product on TG, Cholesterol, HDL and VLDL in male and female rats

Parameters	Males				Females			
	Control	50 mg/kg/day	100 mg/kg/day	200 mg/kg/day	Control	50 mg/kg/day	100 mg/kg/day	200 mg/kg/day
TG	40.4 ± 0.312	41.2 ± 0.845	38.6 ± 0.460	70.08** ± 1.234	40.64 ± 0.456	44.65** ± 0.344	42.72 ± 0.248	72.38** ± 0.733
Cholesterol	68.54 ± 0.275	80.1** ± 0.604	74.72** ± 0.426	57.68** ± 0.303	72.06 ± 0.580	74.01 ± 0.269	74.88** ± 0.620	68.24** ± 0.412
HDL	37.04 ± 0.394	33.96** ± 0.356	22.42** ± 0.290	25.12** ± 0.386	38.72 ± 0.210	35.28** ± 0.397	28.1** ± 0.304	15.64** ± 0.221
VLDL	6.13 ± 0.259	6.14 ± 0.225	6.16 ± 0.263	10.86** ± 0.190	5.86 ± 0.145	5.96 ± 0.128	5.94 ± 0.158	10.96** ± 0.121

The data are expressed as mean and standard error of the mean (*n* = 5). For statistical analysis one-way ANOVA with post-hoc Tukey’s test was applied. * represents significance of *p <* 0.05, ** represents significance of *p <* 0.01

### Histopathological examination of organs

#### Kidney

**Fig. 3:**
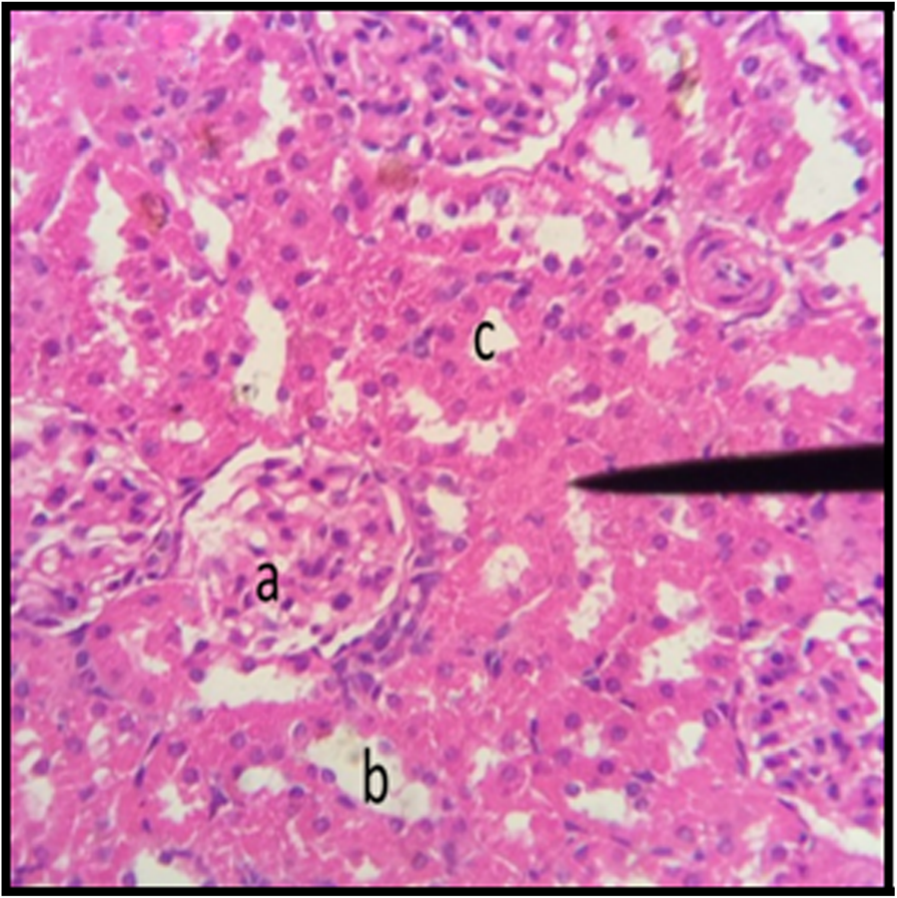
Kidney Section of Male Rat (Control) X400. **a** normal glomerulus of control male rat, **b** normal distal tubules, **c** normal proximal tubules

**Fig. 4:**
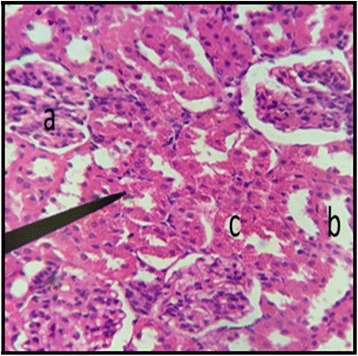
Kidney Section of Female Rat (Control) X400. **a** normal glomerulus, **b** normal distal tubule, **c** normal proximal tubule

**Fig. 5:**
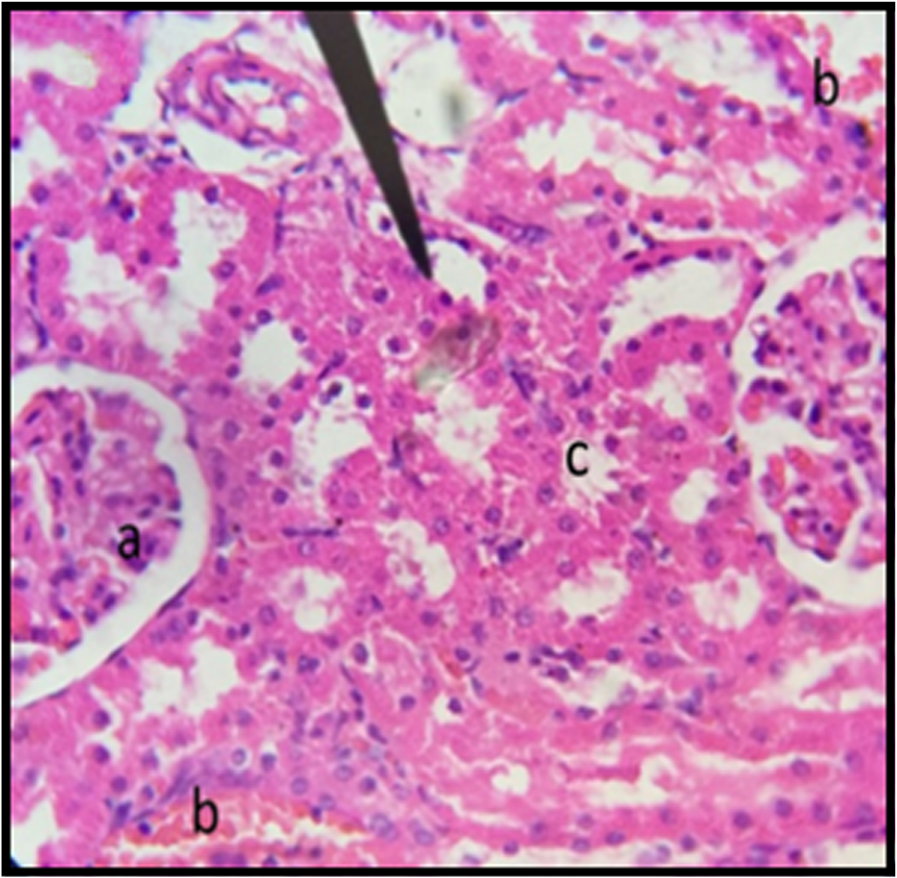
Kidney Section of Male Rat (50 mg/kg/day) X400. **a** normal glomerulus, **b** little bit dilation of peritubular capillaries, **c** normal proximal tubule

**Fig. 6:**
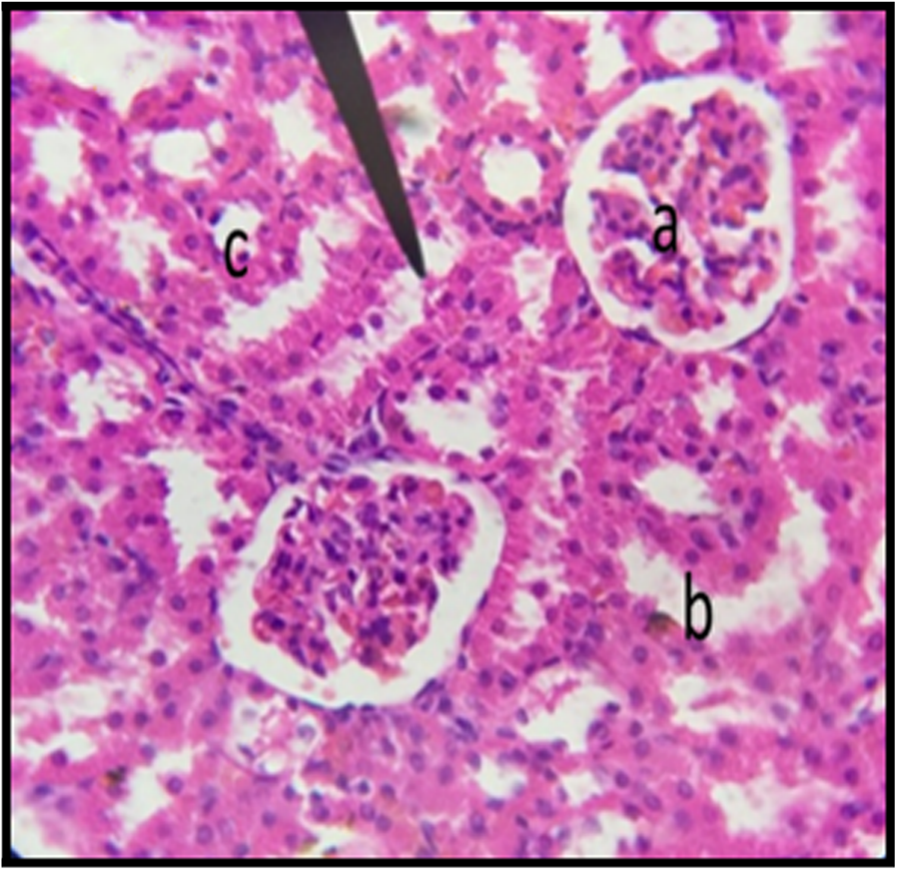
Kidney Section of Female Rat (50 mg/kg/day) X400. **a** normal glomerulus, **b** normal distal tubule, **c** normal proximal tubule

**Fig. 7:**
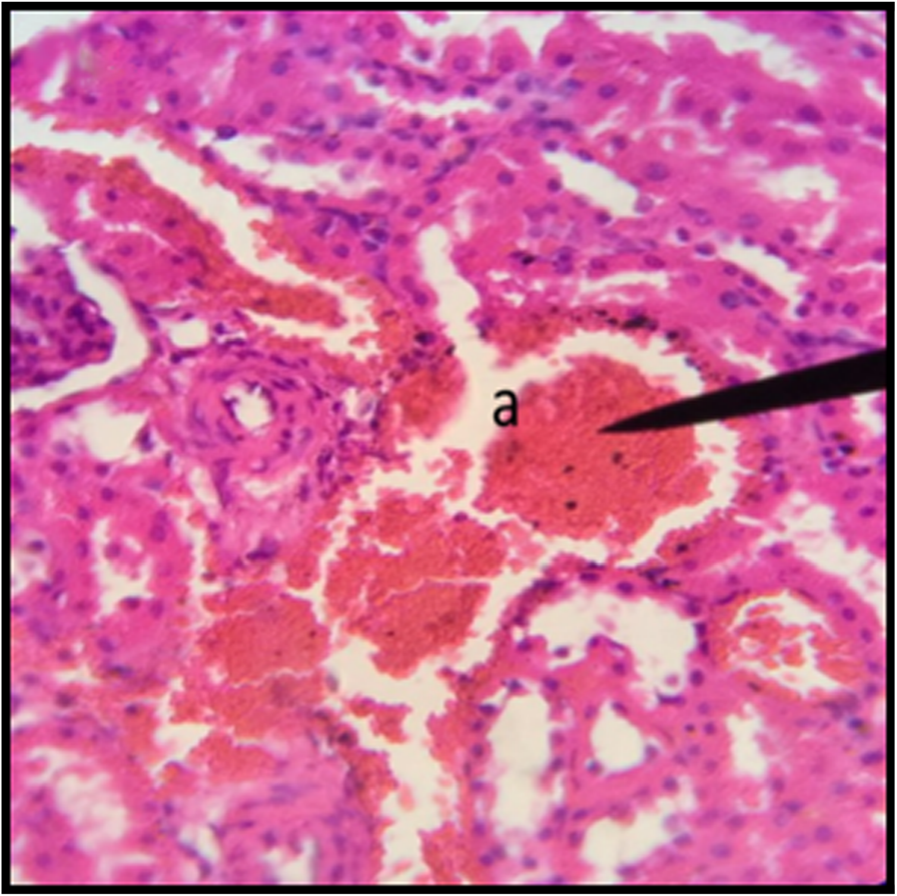
Kidney Section of Male Rat (100 mg/kg/day) X400. **a** coalescence of capillaries along with damage to the glomerulus

**Fig. 8:**
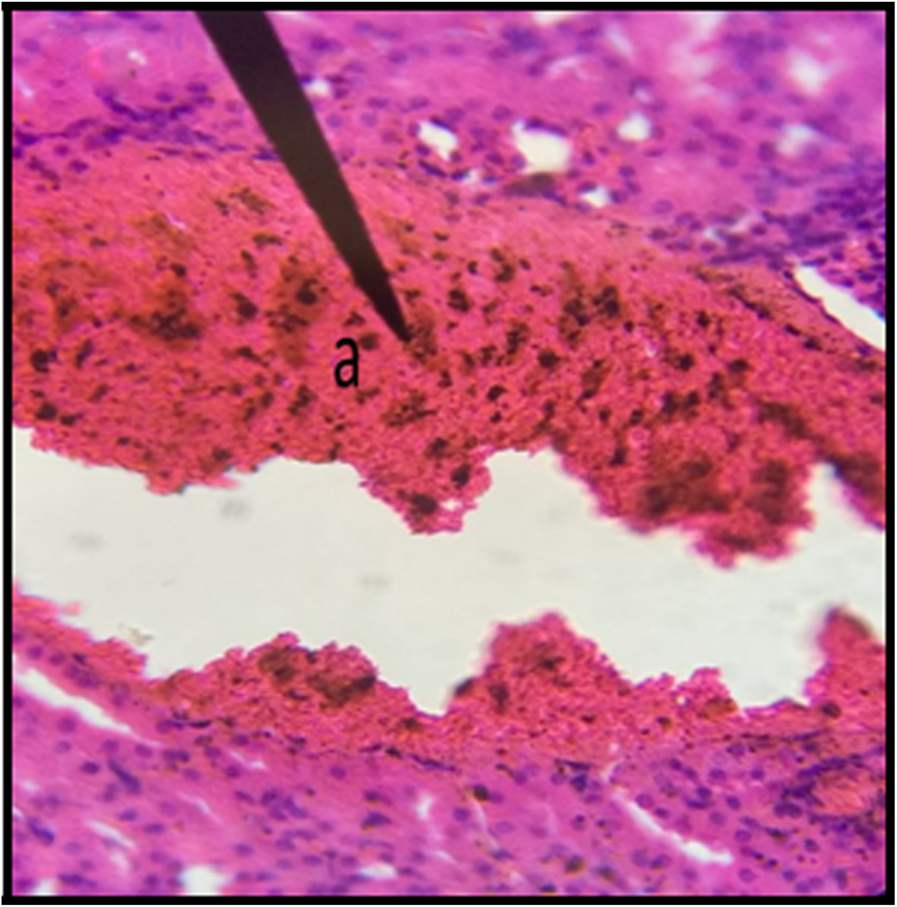
Kidney Section of Female Rat (100 mg/kg/day) X400. **a** damage to nephron along with infiltration of RBCs

**Fig. 9:**
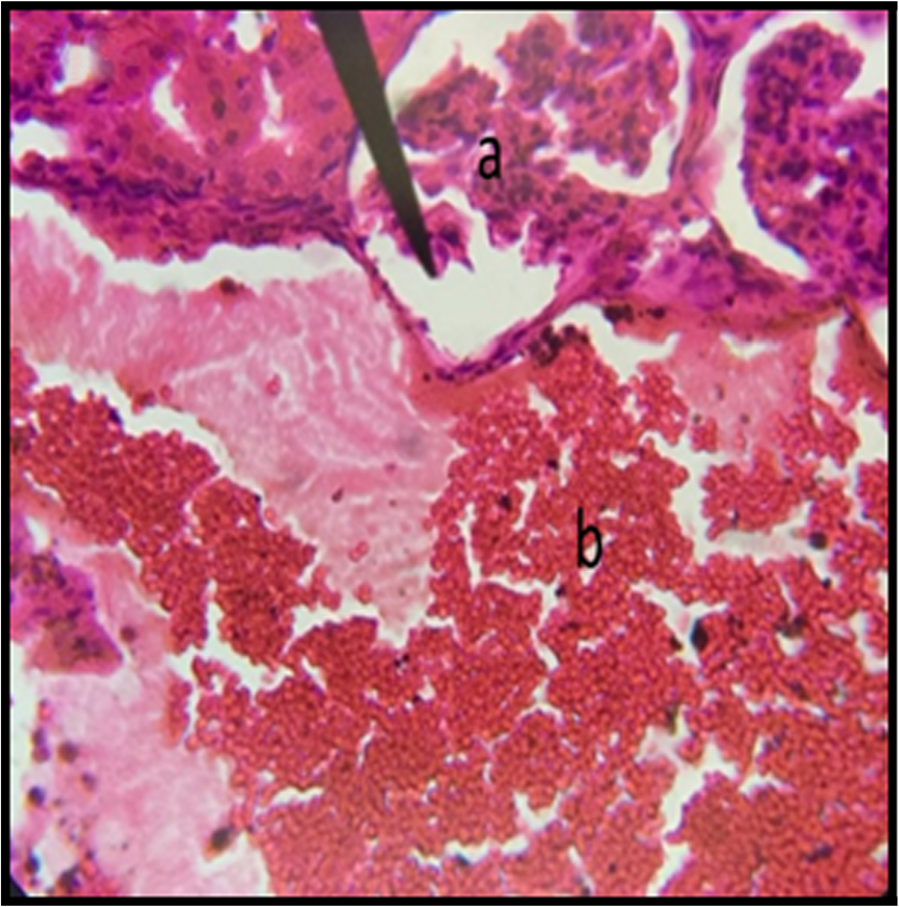
Kidney Section of Male Rat (200 mg/kg/day) X400. **a** normal glomerulus, **b** rupture of the basement membrane along with the infiltration of blood cells

**Fig. 10:**
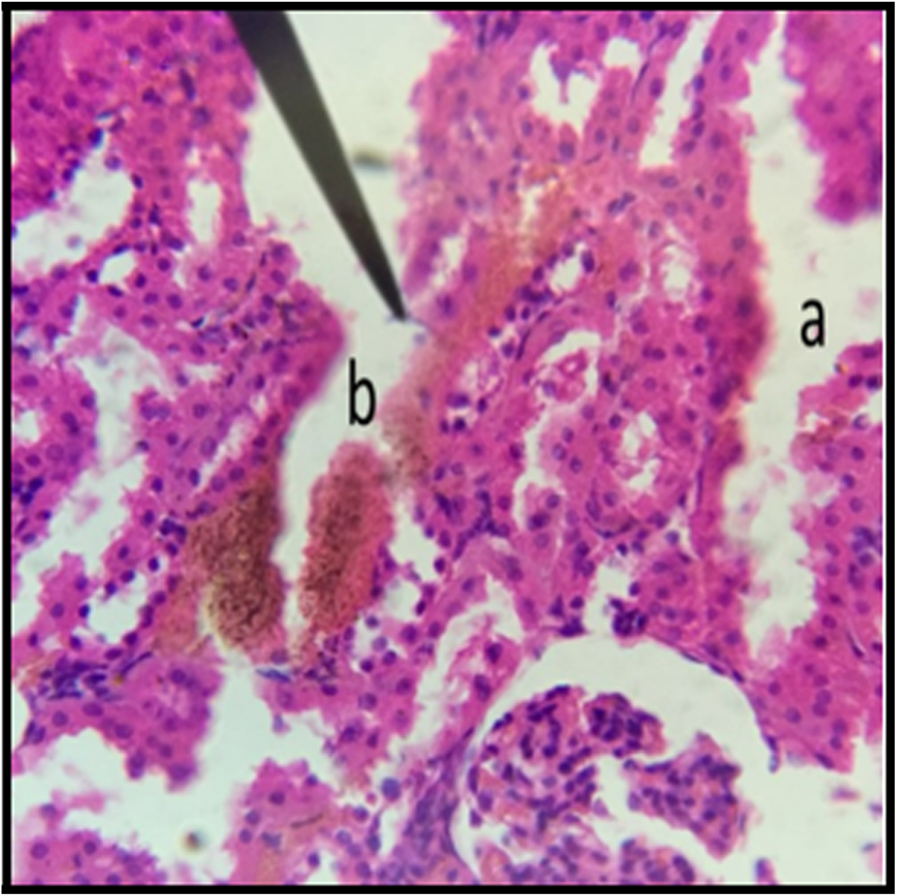
Kidney Section of Female Rat (200 mg/kg/day) X400. **a** congestion of the nephrons, **b** infiltration of the inflammatory cells

The normal distill and proximal tubule along with glomerulus was observed in the slides of male and female rats of control group (Figs. 3 and 4). The histopathological examination of kidney of male and female rats with dose of 50 mg/kg/day showed same symmetry of cells as that of control groups (Figs. 5 and 6) but with doses of 100 mg/kg/day (Figs. 7 and 8) and 200 mg/kg/day (Figs. 9 and 10) showed infiltration of WBCs and RBCs, presented damage in kidney structures of both male and female rats.

### Liver

**Fig. 11:**
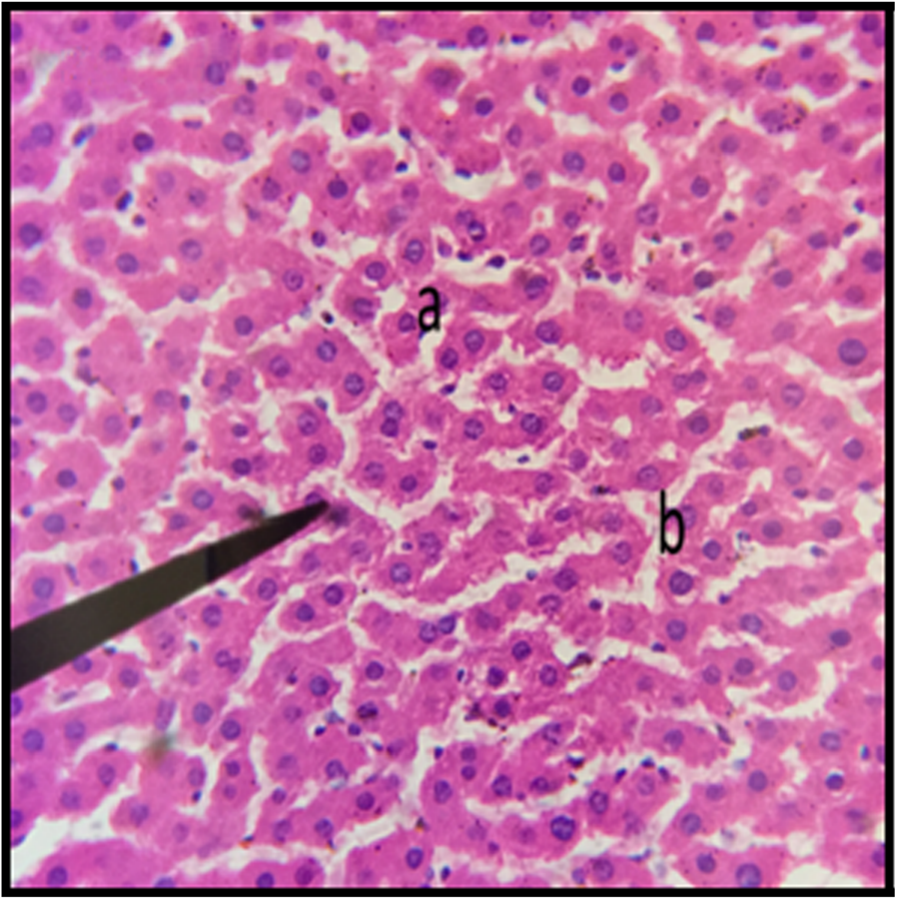
Liver Section of Male Rat (Control) X400. **a** normal sinusoids, **b** central vein, **c:** portal vein

**Fig. 12:**
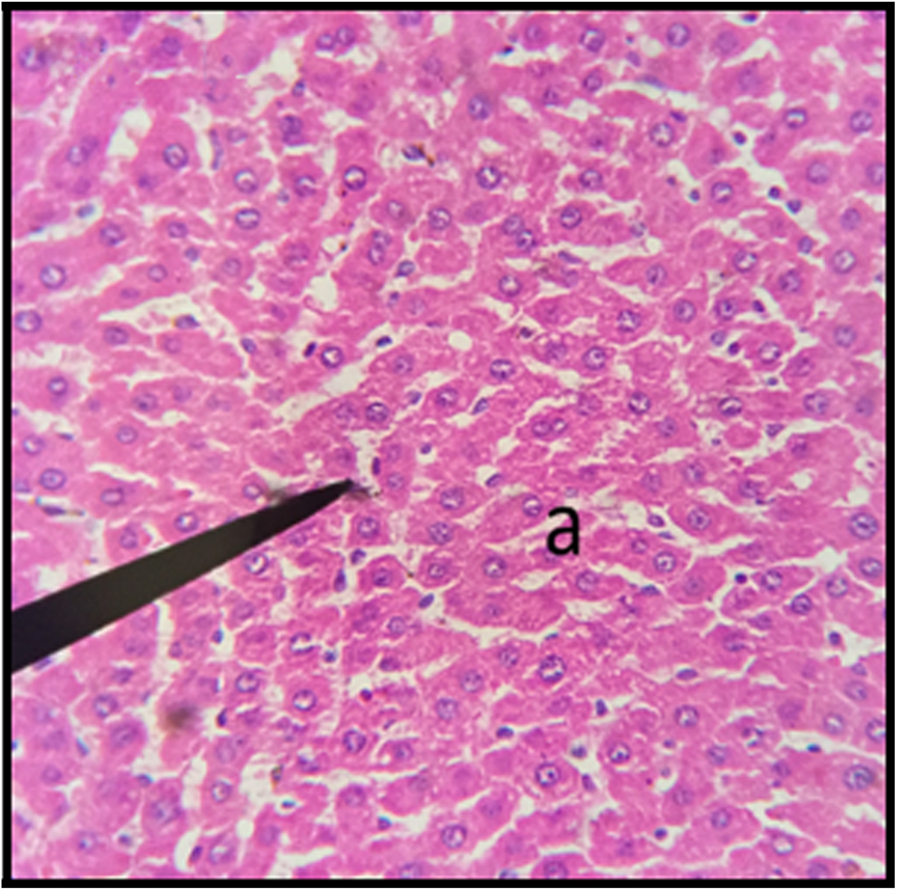
Liver Section of Female Rat (Control) X400. **a** normal sinusoids and hepatocytes

**Fig. 13:**
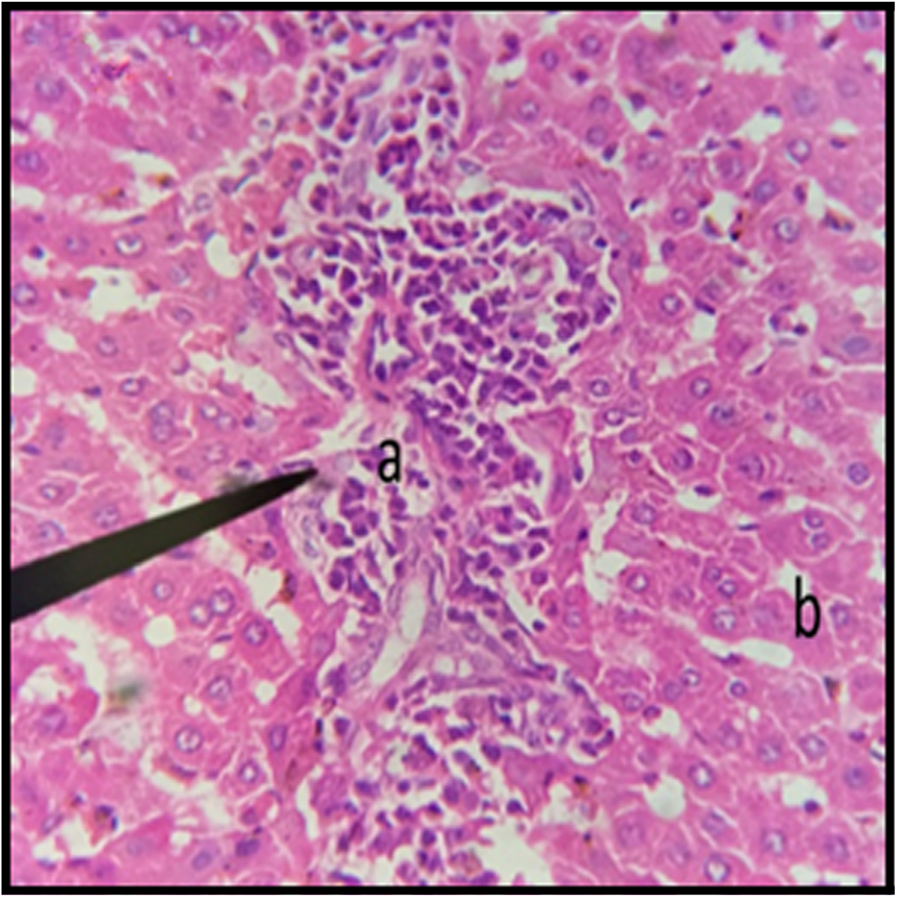
Liver Section of Male Rat (50 mg/kg/day) X400. **a** inflammation in portal vein, **b** normal sinusoids

**Fig. 14:**
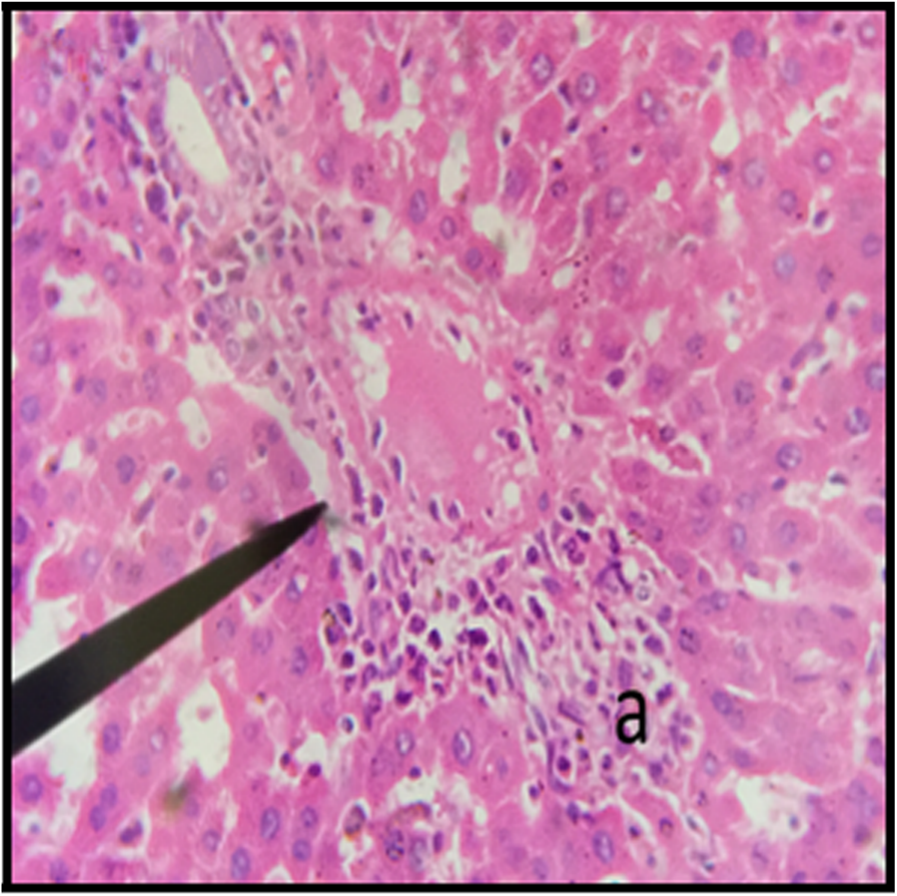
Liver Section of Female Rat (50 mg/kg/day) X400. **a** infiltration of inflammatory cells in sinusoidal spaces

**Fig. 15:**
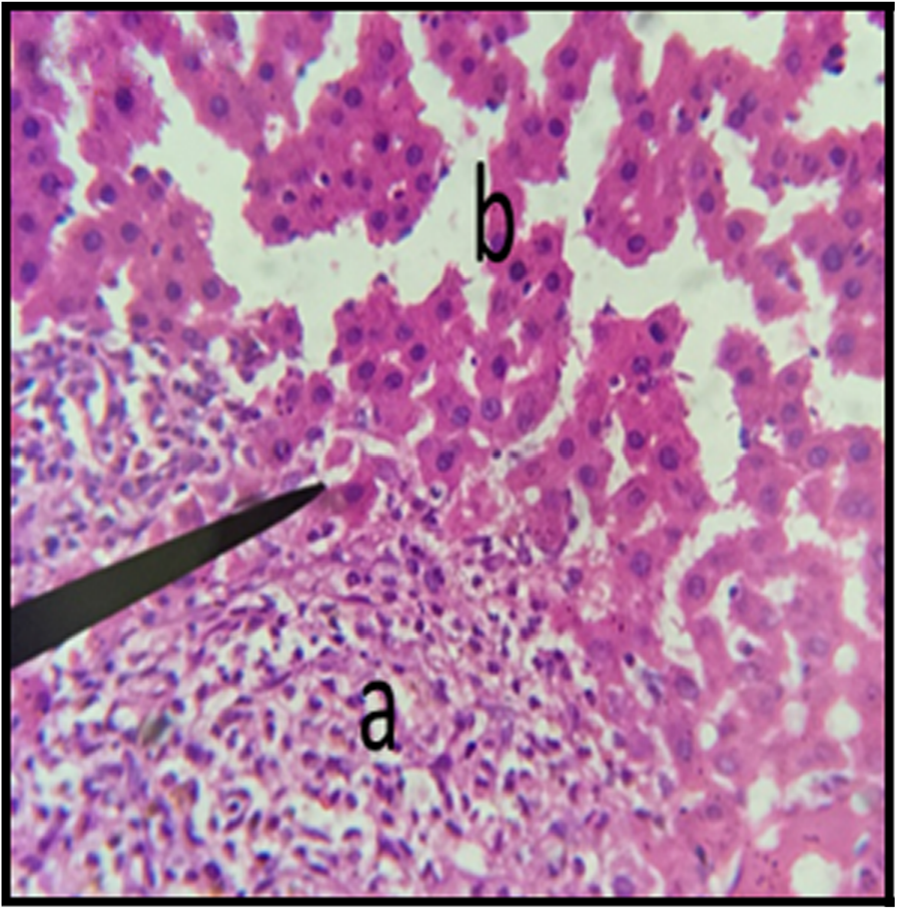
Liver Section of Male Rat (100 mg/kg/day) X400. **a** presence of inflammatory cells, **b** congestion of sinusoids

**Fig. 16:**
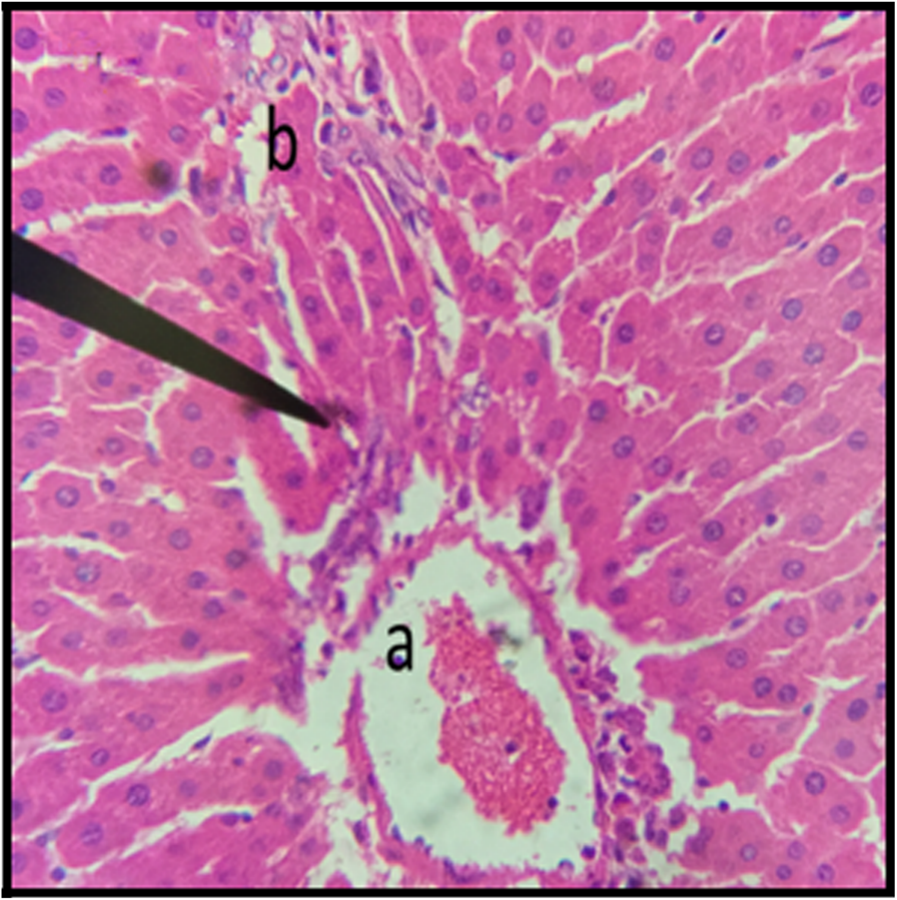
Liver Section of Female Rat (100 mg/kg/day) X400. **a** inflammation of central vein, **b** inflammation surrounding central vein

**Fig. 17:**
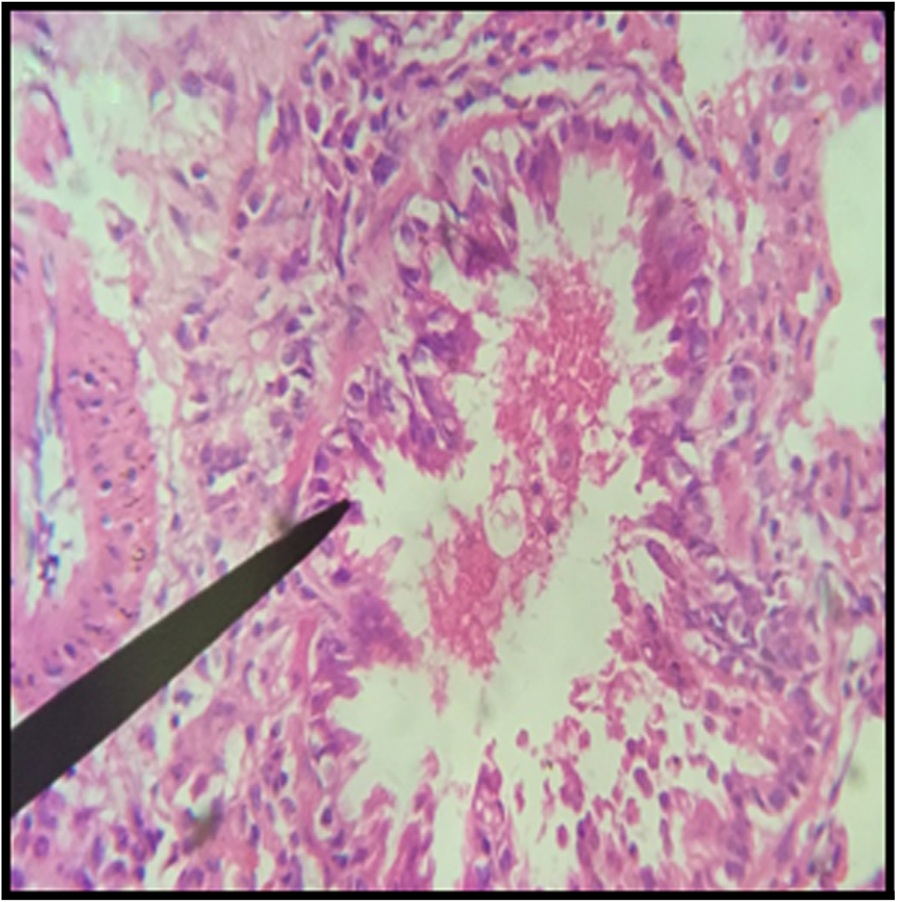
Liver Section of Male Rat (200 mg/kg/day) X400. **a** infiltration of inflammatory cells in central vein, **b** destructive sinusoids

**Fig. 18:**
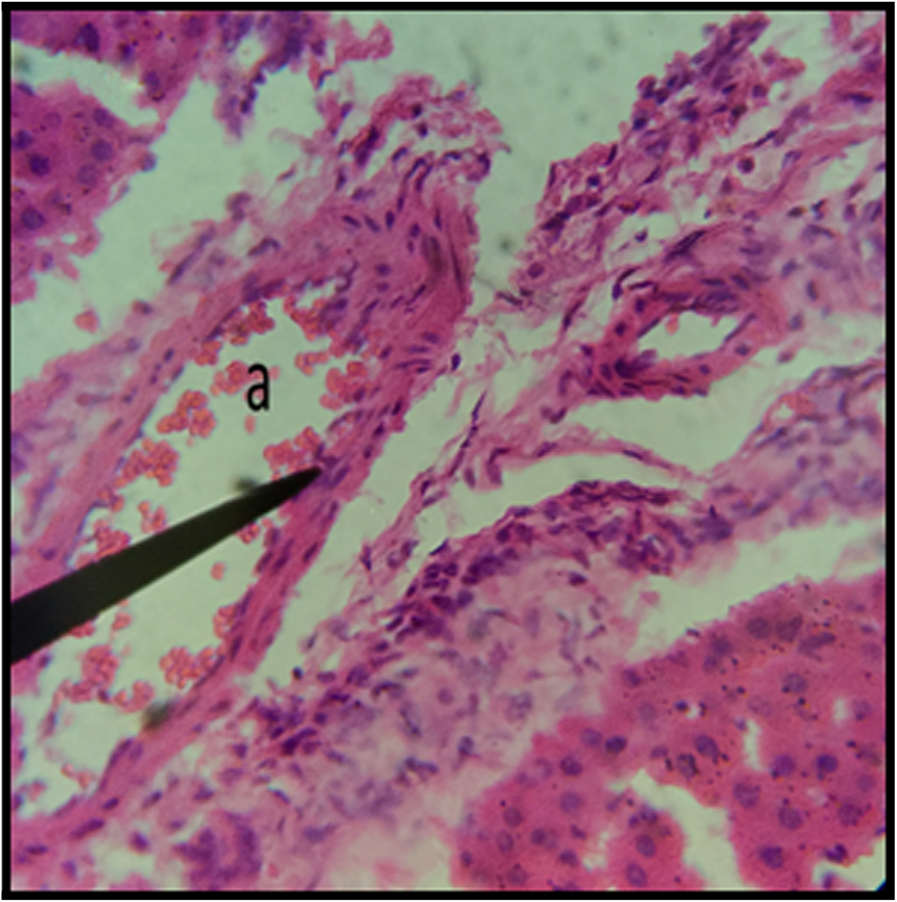
Liver Section of Female Rat (200 mg/kg/day) X400. **a** congestion of hepatocytes

The histopathological examination of liver of both male and female rats with distil water (Figs. 11 and 12) normal structures. The sections with dose of 50 mg/kg/day showed normal structures as that of control group with slight inflammation of portal vein and infiltration of inflammatory cells (Figs. 13 and 14). Male and female rat groups with dose of 100 mg/kg/day showed presence of inflammatory cells and congestion of sinusoids (Figs. 15 and 16), with the subsequent high dose of 200 mg/kg/day, the treatment groups showed the inflammatory cells penetration near central vein and destruction of pattern of sinusoids with congestion of hepatocytes and dilation of sinusoids (Figs. 17 and 18).

## Discussion

Polyherbal formulations are abundantly used in developed countries as compared to allopathic medicine for the treatment of different types of ailments [24]. In Pakistan Hab e Kabad Noshadri tablets, are effectively employed for the treatment of gastric and hepatic ailments. In this study acute and sub-acute toxicity of herbal product was analyzed in male and female Swiss Albino mice and Wistar rats, respectively. The acute toxicity study was conducted for 72 h. at the dose of 2000 mg/kg/day, no morbidity and mortality was observed. This proves that the drug could be safely administered for acute treatments up to the dose of 2000 mg/kg/day.

During the sub-acute toxicity study non-significant changes in hematological and biochemical parameters of liver, lipids and renal markers were observed with 50 mg/kg/day and 100 mg/kg/day depicting that these doses could be safely employed for the treatment of liver disorders as claimed by the label. The label claims presence of *Embelia* and *Senna* as an ingredients in the formulation that have proved to be effective in liver disorders [25, 26]. The formulation also contain chebulicmyrobalan yellow *(Terminalia chebula)*, that has phenolic content which inhibits phenotype change in HSC in liver and reduces the infiltration of neutrophils in the liver and protecting liver from inflammational damage [27], as observed at dose of 50 mg/kg/day during sub-acute study. The formulation also contains zedoary *(Curcuma zedoaria),* ginger *(Zingiber officinale)*, senna leaflets *(Cassia angustifolia, Cassia acutifolia),* belericmyrobalan *(Terminalia belerica),* black pepper *(Piper nigrum),* and chebulicmyrobalan yellow *(Terminalia chebula)* reported to possess anti-inflammatory actions. These drugs have been reported to possess different types of flavonoids, therefore exhibiting anti-inflammatory actions when given in combination [27–34]. Belericmyrobalan *(Terminalia belerica)* inhibits the inflammatory mediators and chemotactic cytokines (TNFα and IL-6) and decreases inflammatory reactions [35]. Black pepper *(Piper nigrum)*, contains piperine responsible for antioxidant anti-inflammatory action that hinders in many signaling pathways that may involve in the proliferation of T-lymphocytes and thus protecting body from many chronic inflammatory diseases [36]. The formulation also contains chebulicmyrobalan black *(Terminalia chebula)* analyzed by researcher, possessing kidney protective action [37] also proved in our study at dose of 50 mg/kg/day and the results are presented in Table 4. Embelia *(Embelia ribs)* has strong ability on dyslipidemia associated with hypercholesterolemia. It reduces cholesterol and TG levels [38] at dose of 50 mg/kg/day presented in Table 5. Belericmyrobalan *(Terminalia belerica)* increases body weight and decreases serum cholesterol, triglycerides, uric acid and creatinine [39] that was also observed during sub-acute toxicity study at dose of 50 mg/kg/day in both male and female rats (Figs. 1 and 2).

In the histopathalogical examination no changes in liver and kidney morphology were seen at dose of 50 mg/kg/day whereas, congestion with mild inflammation was observed due to the infiltration of neutrophils in the liver with 100 mg/kg/day. The kidney’s histopathological examination also showed coalescence of capillaries with the infiltration of RBCs suggesting that the dose of 100 mg/kg/day should be cautiously prescribed by the practitioners. However, no change in the biochemical parameters was observed during sub-acute study but the structural changes at 100 mg/kg/day suggests that the product is more appropriate to be prescribed at dose of 50 mg/kg/day.

During the sub-acute toxicity studies, a significant decrease in the liver enzymes with 200 mg/kg/day was observed in both male and female rats and this decrease was below the normal reference range. The bilirubin levels were also significantly raised in female rats. It was due to the non-functional behavior of the hepatocytes in the liver that happened due to the coalescence of the hepatocytes. In the histopathological observation of liver the shrinkage (atrophy) and fusion of the hepatocytes was observed (Figs. 17 and 18), and the highly significant (*p* < 0.01) decrease in the liver enzymes (Table 5) suggests that 200 mg/kg/day should not be prescribed. The physical abnormalities viz. nasal bleeding on 17th and 18th day and right upper paw paralysis after 15 days of treatment was also observed during the sub-acute study. The nasal bleeding may be due to the decreased synthesis of plasma proteins by the destructed liver that are responsible for the clotting [40, 41]. Liver damage also results into portal hypertension which consequently shows the complication of bleeding due to the raised pressure [42]. Similarly, the compromising renal and ESRD also results in hemostatic disorders mainly in the form of bleeding diatheses [43]. On the other hand the right upper paw paralysis may be due to the neurodegenerative action of the formulation in the specific part of brain controlling the right upper paw of the test subjects. These clinical signs suggests that 200 mg/kg/day is not safe to be prescribed by the healthcare providers. The levels of urea were significantly increased in both male and female rats, uric acid was also significantly raised in female treated group. Uremia (increased urea in blood) and hyperurecemia (increased uric acid in blood) indicates the nephrotoxic potential of the formulation at dose of 200 mg/kg/day. The histopathological examination of the kidney also showed damage (Figs. 9 and 10) to the nephrons suggesting that prolonged use of formulation may result into CKD. The levels of TG and VLDL were increased in both male and female rats whereas, HDL was significantly decreased suggesting disorders in lipid metabolism which may lead to cardiovascular diseases. Significant increase in the WBCs and PLT count with 100 and 200 mg/kg/day also proves that these doses result into an increase in the inflammatory burden. It could be suggested that oriental herbalogy may result into interactions or toxicity on prolong use, when given in higher doses.

## Conclusions

The findings of acute study revealed that this polyherbal formulation is non-toxic with single oral dose of 2000 mg/kg/day. The 28 days sub-acute toxicity study, revealed no significant changes with 50 mg/kg/day. Slight changes in biochemical parameters and structural levels was at 100 mg/kg/day and severe cellular changes at 200 mg/kg/day. So, it is concluded that the formulation is safe to use at dose of 50 mg/kg/day for a period of 28 days whereas the 100 mg/kg/day should be cautiously employed and 200 mg/kg/day should not be recommended. In future it is recommended to analyze the effects of the individual ingredients on the organs and tissues at different doses.

### Supplementary material

The doses are converted according to the following chart [44] Table 7.

**Table 7:** Human equivalent dose calculation based on body surface area^a^

Species	References body weight (kg)	References body range (kg)	Body surface area (m^2^)	To convert dose in mg/kg to dose in mg/m^2^, multiply by K_m_	To convert animal dose in mg/kg, either	
					Divide animal dose by	Divide animal dose by
Human	60	-	1.62	37	-	-
Mouse	0.02	0.011-0.3034	0.007	3	12.3	0.081
Hamster	0.08	0.047-0.157	0.016	5	7.4	0.135
Rat	0.15	0.08-0.27	0.025	6	6.2	0.162
Ferret	0.20	0.16-0.54	0.043	7	5.3	0.189
Guinea pig	0.40	0.208-0.700	0.05	8	4.6	0.216
Rabbit	1.8	0.90-3.0	0.15	12	3.1	0.324
Dog	10	5-17	0.50	20	1.8	0.541
Monkey (rhesus)	3	1.4-4.9	0.25	12	3.1	0.324
Marmoset	0.35	0.14-0.72	0.06	6	6.2	0.162
Squirrel monkey	0.60	0.29-0.97	0.09	7	5.3	0.189
Baboon	12	7-23	0.60	20	1.8	0.541
Macro pig	20	10-23	0.74	27	1.4	0.730
Mini pig	40	25.64	1.14	35	1.1	0.946

^a^Data obtained from FDA draft guidelines.^[7]^ FDA: Food and Drug Admin istration, HED: Human equivalent dose

Dose 1 = 50 mg/kg/day.

Dose for 0.15 kg rat = 50 × 0.15 = 7.5 mg/ 0.15 kg.

Conversion into Human equivalent dose (HED) = 7.5 × 0.162 = 1.215 mg/kg.

Dose for 60 kg (Avg.) human = 1.215 × 60 = 72.9 mg/ 60 kg man ≈ 75 mg / 60 kg.

Dose 2 = 100 mg/kg/day.

Dose for 0.15 kg rat = 100 × 0.15 = 15 mg/ 0.15 kg.

Conversion into Human equivalent dose (HED) = 15 × 0.162 = 2.43 mg/kg.

Dose for 60 kg (Avg.) human = 2.43 × 60 = 145.8 mg/ 60 kg man ≈ 150 mg / 60 kg.

Dose 3 = 200 mg/kg/day.

Dose for 0.15 kg rat = 200 × 0.15 = 30 mg/ 0.15 kg.

Conversion into Human equivalent dose (HED) = 30 × 0.162 = 4.86 mg/kg.

Dose for 60 kg (Avg.) human = 4.86 × 60 = 291.6 mg/ 60 kg man ≈ 300 mg / 60 kg.

According to product’s regimen = 2 Tab. TID (Wt. of Tab. is 500 mg), it means 6 tablets a day.

So, 50 mg/kg = 75 × 6 = 450 mg ≈ 500 mg / 60 kg / day.

100 mg/kg = 150 × 6 = 900 mg ≈ 1000 mg / 60 kg / day.

200 mg/kg = 300 × 6 = 1800 mg ≈ 2000 mg / 60 kg / day.

The clinically used dose i.e. 3 g doesn’t appear to be safe. As from our study it was seen that the margin of safety is narrow that’s why the conclusion was made to prescribe 100 mg/kg cautiously to the patients unless otherwise the therapy should be continued with 50 mg/kg.

## Additional file

Additional file 1: Historical Control Ranges for Selected Clinical Chemestry Parameters in Laboratory Animals. (JPEG 143 kb)

## Abbreviations

ALT: Alanine transaminase
AST: Aspartate transaminase
CKD: Chronic kidney disease
EDTA: Ethylenediamine tetra acetate
ESRD: End stage renal disease
h: hour
Hb: Hemoglobin
HCT: Hematocrit
HDL: High density lipoprotein
HED: Human equivalent dose.
hrs.: hours
HSC: Hepatic stellate cells
LD_50_: Lethal dose
MCH: Mean cell hemoglobin
MCHC: Mean cell hemoglobin concentration
MCV: Mean corpuscular volume
NOEL: Non observable effect level
p.o.: Per-oral
PLT: Platelet
RBCs: Red blood cells
TG: Triglycerides
VLDL: Very low density lipoprotein
WBCs: White blood cells
