# Supplementary figures and images for: Acute and sub-acute toxicity study of a Pakistani polyherbal formulation
Source: BMC Complement Altern Med. 2017 Aug 4;17:387. doi: 10.1186/s12906-017-1889-7 (PMC5545041; doi:10.1186/s12906-017-1889-7)

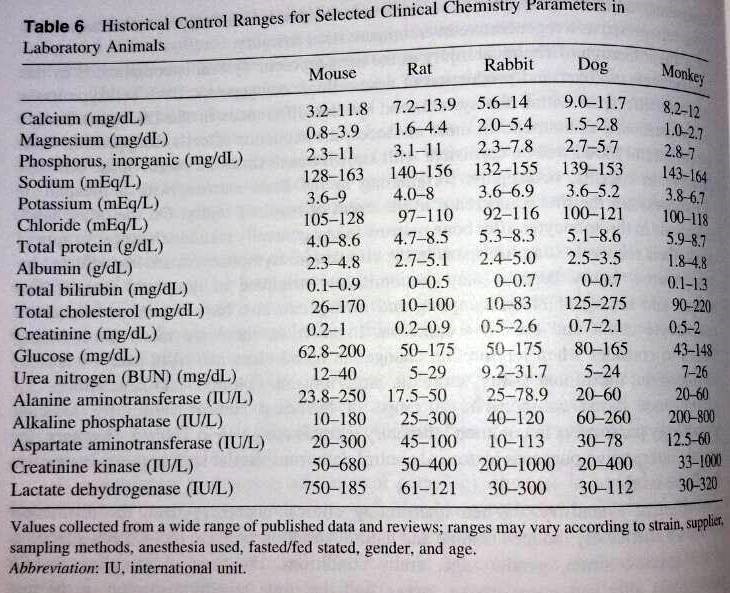

Supplement: Additional file 1: — Historical Control Ranges for Selected Clinical Chemestry Parameters in Laboratory Animals. (JPEG 143 kb) [file 12906_2017_1889_MOESM1_ESM.jpg]
